# Supplementary material for: Disease-associated mutations within the yeast DNAJB6 homolog Sis1 slow conformer-specific substrate processing and can be corrected by the modulation of nucleotide exchange factors
Source: Nat Commun. 2022 Aug 5;13:4570. doi: 10.1038/s41467-022-32318-9 (PMC9355953; doi:10.1038/s41467-022-32318-9)
Supplement: Supplementary file 1 — Supplementary information file [file 41467_2022_32318_MOESM1_ESM.pdf]

Disease-associated mutations within the yeast DNAJB6 homolog Sis1 slow conformer-specific substrate processing and can be corrected by the modulation of nucleotide exchange factors

Ankan K. Bhadra[1], Michael J. Rau[2], Jil A. Daw[4], James A.J. Fitzpatrick[1, 2, 3], Conrad C. Weihl[4], Heather L. True[1*]

1. Department of Cell Biology and Physiology, Washington University School of Medicine, 660 South Euclid Avenue, Campus Box 8228, St. Louis, MO, 63110, USA. Electronic address: heather.true@wustl.edu.

2. Washington University Center for Cellular Imaging (WUCCI), Washington University School of Medicine, St. Louis, United States.

3. Department of Neuroscience, Washington University School of Medicine, St. Louis, United States.

4. Department of Neurology, Hope Center for Neurological Diseases, Washington University School of Medicine, St. Louis, MO, USA.

Supplementary information includes 10 figures, 1 table and supplementary figure references.

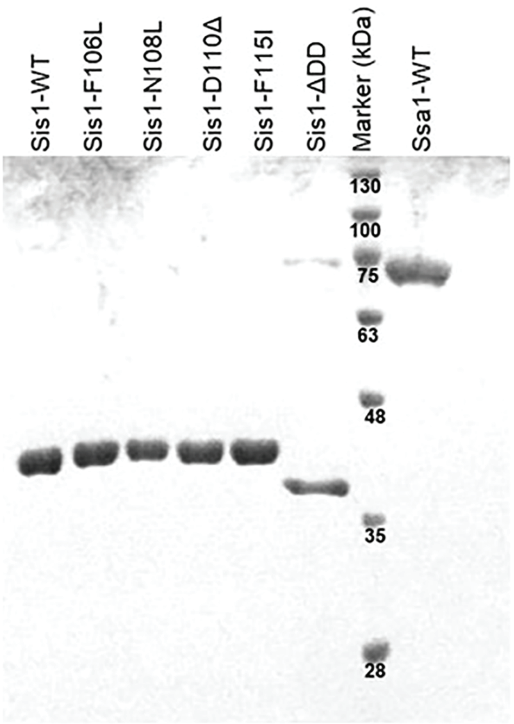

Supplementary Fig. 1. SDS-PAGE showing the image of purified protein bands for Sis1-WT, Sis1-F106L, Sis1-N108L, Sis1-D110Δ, Sis1-F115I, Sis1-ΔDD, Marker, Ssa1-WT (from left to right) used in assays. One representative image from n=3 is shown here.

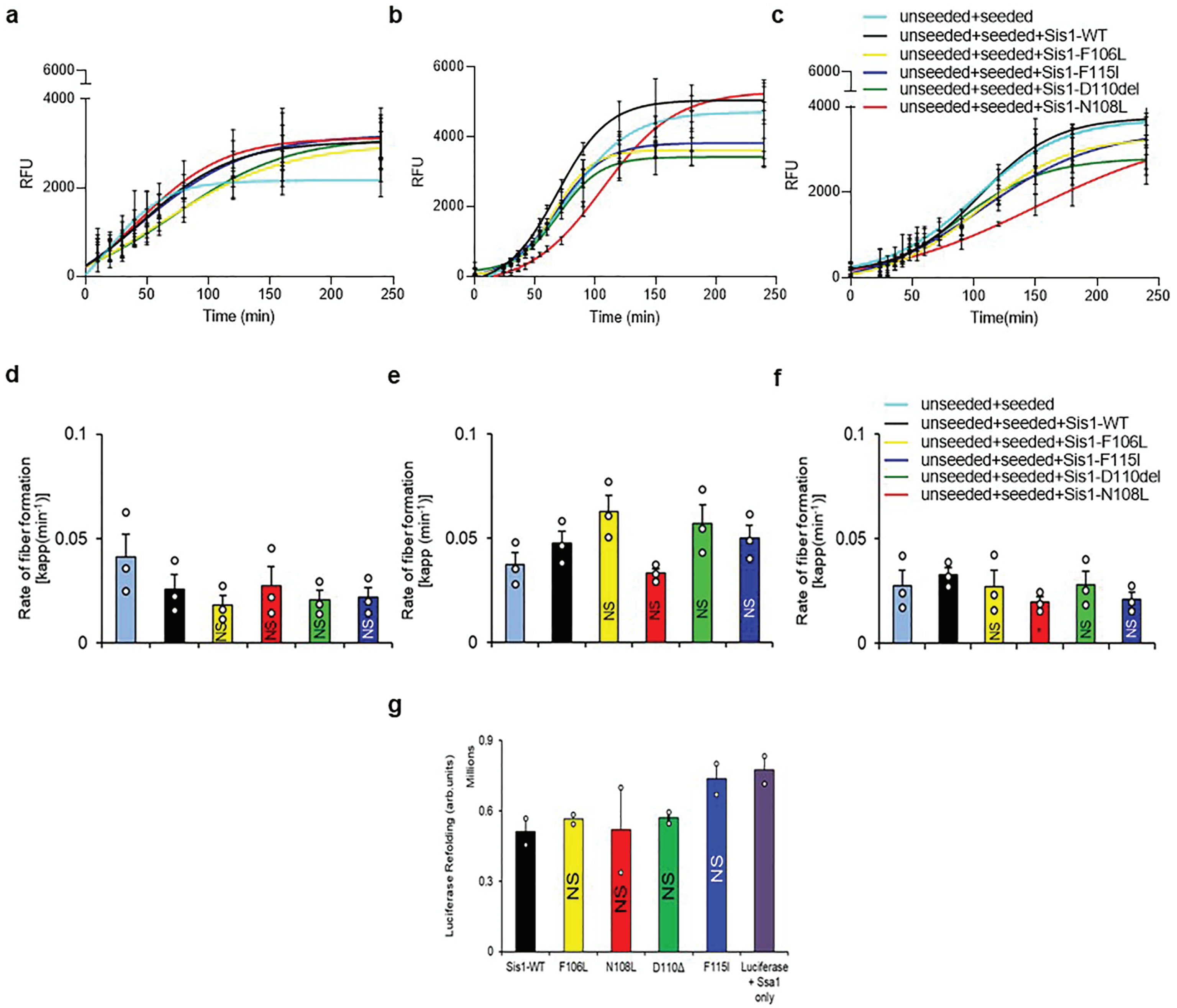

Supplementary Fig. 2. **LGMDD1 G/F domain mutants behave differentially in aggregation assays with preformed Rnq1 seeds**. (a), (b), and (c) Kinetics of Rnq1 fibrillation in the presence of unseeded and seeded Rnq1 only (cyan blue), Sis1-WT (black), Sis1-F106L (yellow), Sis1-N108L (red), Sis1-D110Δ (green), or Sis1-F115I (blue) was measured by ThT fluorescence assay. Note the abrupt increase in the fluorescence intensity of ThT indicates the formation of Rnq1 aggregates. Additionally, the fluorescence intensity of the dye achieved a plateau, indicating the stationary phase in fibrillation of Rnq1. *Inset*:-Fitted graph using the aggregation kinetics equation $y=yi+mxi+(yf+mxf)/(1+e^{x0/\tau})$ where ($yi + mxi$) is the initial line, ($yf + mxf$) is the final line and x0 is the midpoint of maximum signal. 10 % of seeds (v/v) formed at different temperatures 18°C, 25°C, and 37°C were used in the assay. Data represented as mean ± SEM, n=3 biologically independent samples. For (d), (e), and (f) the apparent rate constant (kapp) of Rnq1 aggregation (rate of fibril formation) was measured by calculating 1/τ from the same fitting equation. Note that there was no difference in the rate of fibril formation (in the presence of pre-formed fibrils) between Sis1-WT vs. LGMDD1 mutants at any temperatures. Values shown here are calculated from (a), (b), and (c) respectively. In all cases, LGMDD1 mutants were compared against Sis1-WT; *$p<0.05$, NS (non-significant) values are reported for unpaired, two-sided *t*-test. (g) Graph showing the refolding activity of heat-denatured luciferase at time (t=5 min) in the presence of LGMDD1 mutants. Note that there was no difference in luciferase activity observed with any of mutants when compared with Sis1-WT at time (t=5 min) indicating that the major effect was not on the extent of luciferase inactivation. Values shown are mean + SEM, n=2 biologically independent samples. In all cases, LGMDD1 mutants were compared against Sis1-WT; NS (non-significant) values are reported for unpaired, two-sided *t*-test. Source data are provided as a Source data file.

a

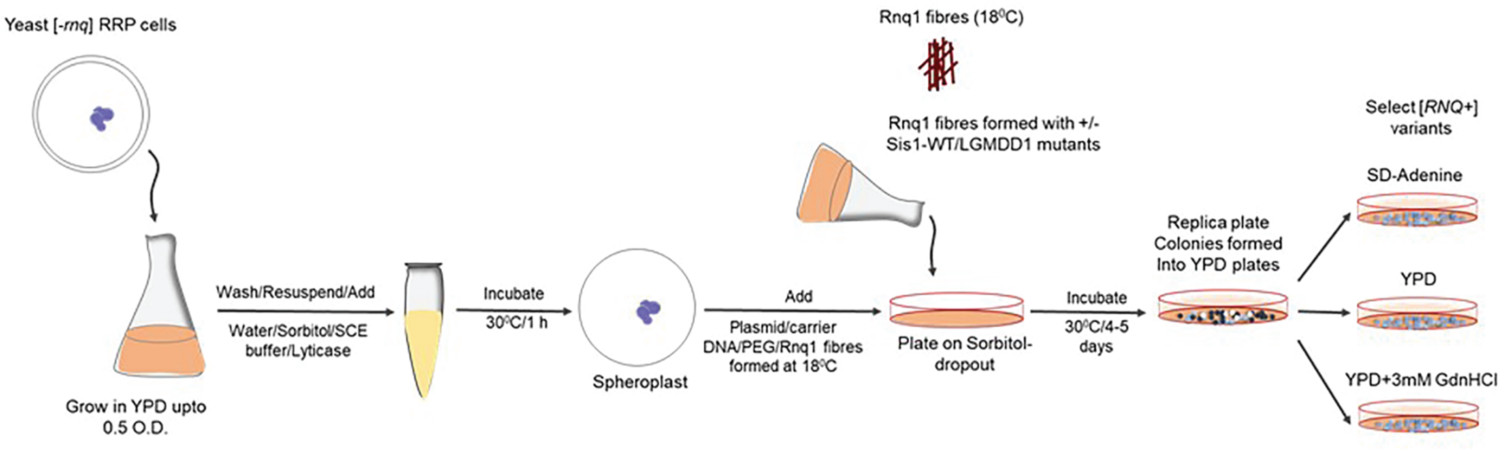

b

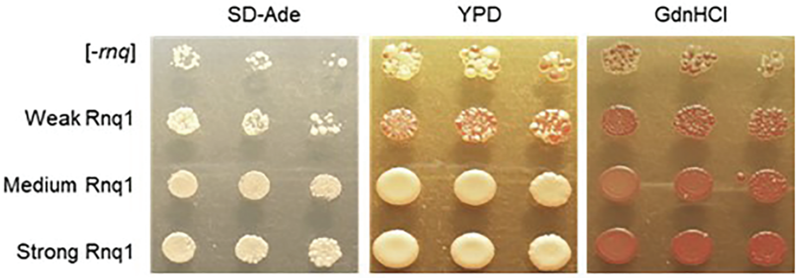

Supplementary Fig. 3. **The protein fiber transformation assay and the prion strain scoring method.** (a) Cartoon depicting the detailed methodology as to how the assay was performed. We took advantage of our [*RNQ+*] reporter protein (RRP) to phenotypically assess [*RNQ+*] prion variants[1]. RRP is a chimeric protein with the PFD of Rnq1 (amino acids 153–405) fused to the translation termination domain of Sup35 (amino acids 124–685)[2]. RRP co-aggregates with Rnq1 in the [*RNQ+*] state, resulting in prion-dependent nonsense suppression and thereby providing a phenotypic assay for the [*RNQ*] state. The level of nonsense suppression afforded by RRP changes with [*RNQ+*] prion variants, enabling us to distinguish variants by phenotype (b). The 74-D694 yeast strain harbors an adenine mutation (*ade1-14*) and this leads to red coloration of colonies grown in rich medium (YPD) that are efficient in translation termination. The aggregation of RRP in different [*RNQ+*] variants result in different levels of suppression of the *ade1-14* premature stop codon and corresponding changes in the production of Ade1.

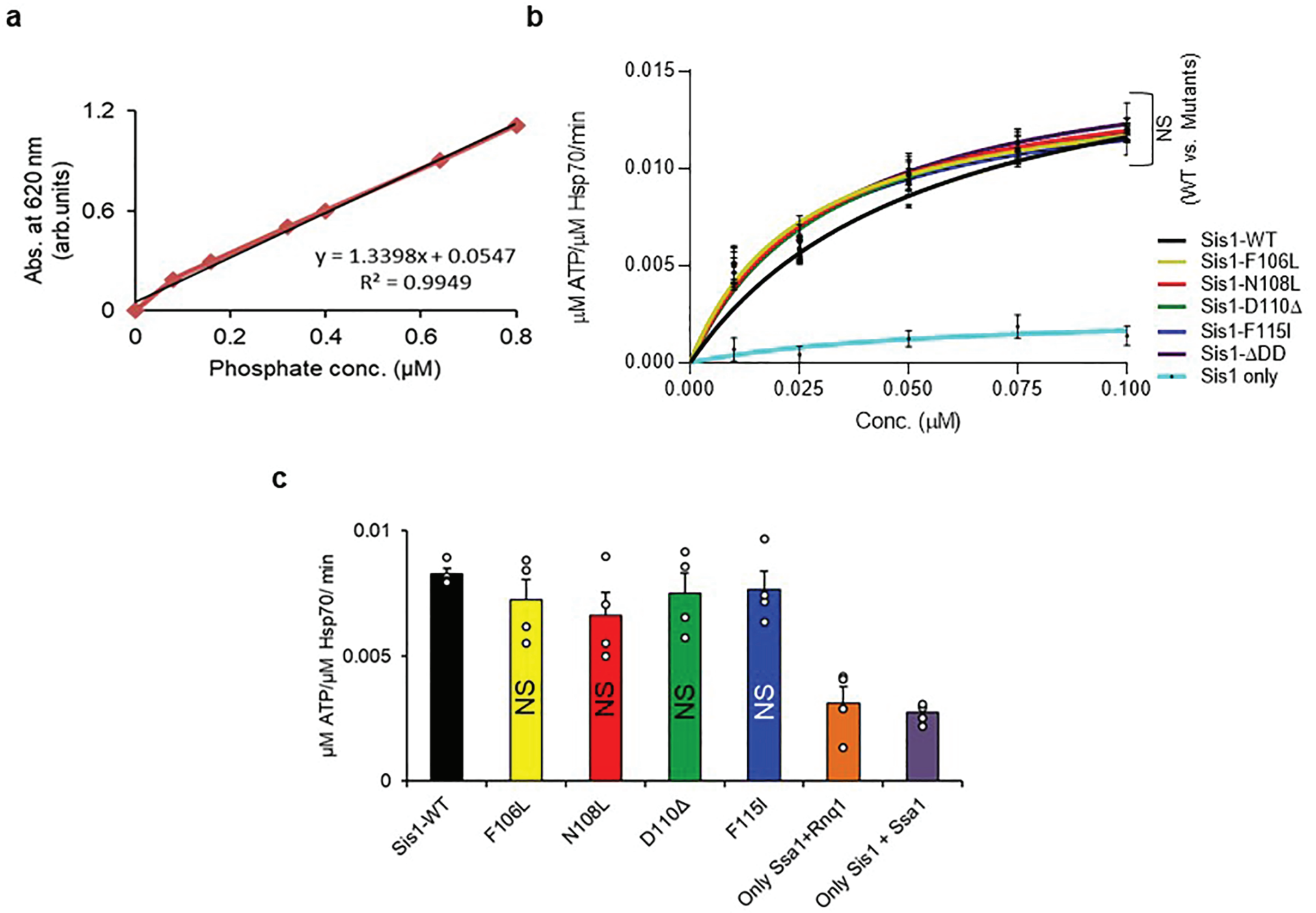

Supplementary Fig. 4. (a) Representative Phosphate Standard Curve. ATPase assay in absence of client protein (b) and in the presence of Rnq1 monomer (c). For (b); varying concentration of Sis1-WT (0.01µM-0.1µM) was incubated with Ssa1 (1µM) and ATP (1mM). Data represented as mean ± SEM, n=3 biologically independent samples. Sis1-WT was compared with LGMDD1- mutants and the differences were non-significant (NS) are reported for unpaired, two-sided *t*-test. For (c); Sis1-WT or mutants (0.05 µM) and Ssa1 (1 µM) combined with ATP (1 mM). For (c) Rnq1 monomer used was 25 µM. Values shown are mean + SEM, n=4 biologically independent samples. All LGMDD1 mutants were compared with Sis1-WT; NS (non-significant) values (from left to right; NS=0.266, NS=0.13, NS=0.398, NS=0.448) are reported for unpaired, two-sided *t*-test. Source data are provided as a Source data file.

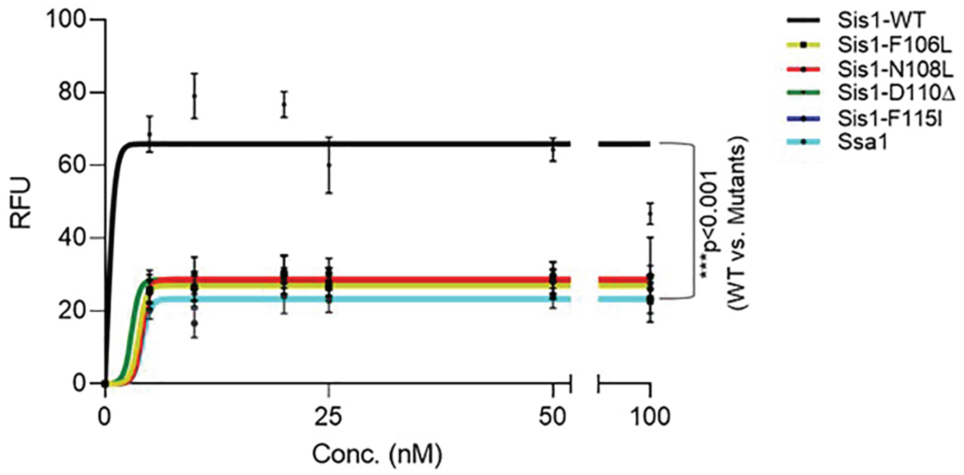

Supplementary Fig. 5. **Sis1 binding to Hsp70 is compromised in the presence of LGMDD1 G/F domain mutants**. Denatured luciferase were premixed with Sis1-WT/mutants and immobilized in microtiter plate wells and dilutions of Ssa1-WT (0-100 nM) were incubated with it. Bound Ssa1-WT was detected using an αSsa1 antibody. Only Ssa1 (cyan blue) was used as a control. Data represented as mean values ± SEM, n=3 biologically independent samples. Sis1-WT was compared to LGMDD1 mutants and ***$p<0.001$ values are reported for unpaired, two-sided *t*-test. RFU stands for Relative fluorescence unit. Source data are provided as a Source data file.

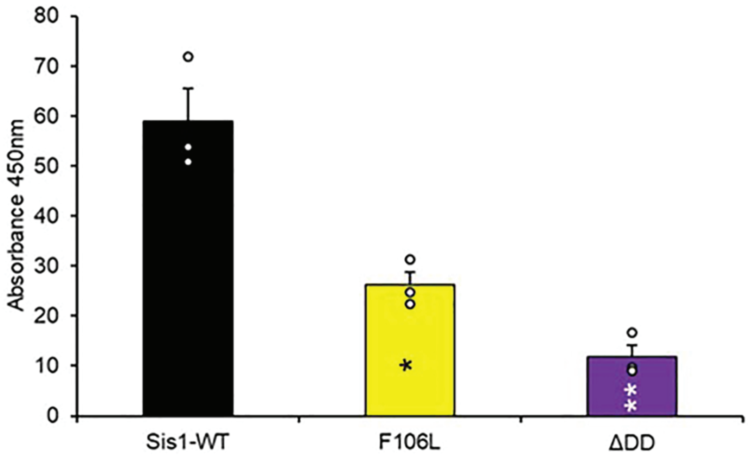

Supplementary Fig. 6. **Absence of Sis1-dimerization domain (Sis1-ΔDD) reduced dimerization.** The Sis1-ΔDD protein was used as a control to validate the assay for dimerization. Data represented as mean + SEM, n=3 biologically independent samples. Mutants were compared with Sis1-WT; ***p*<0.002 (for ΔDD), and **p*<0.02 (for F106L) values are reported for unpaired, two-sided *t*-test. Source data are provided as a Source data file.

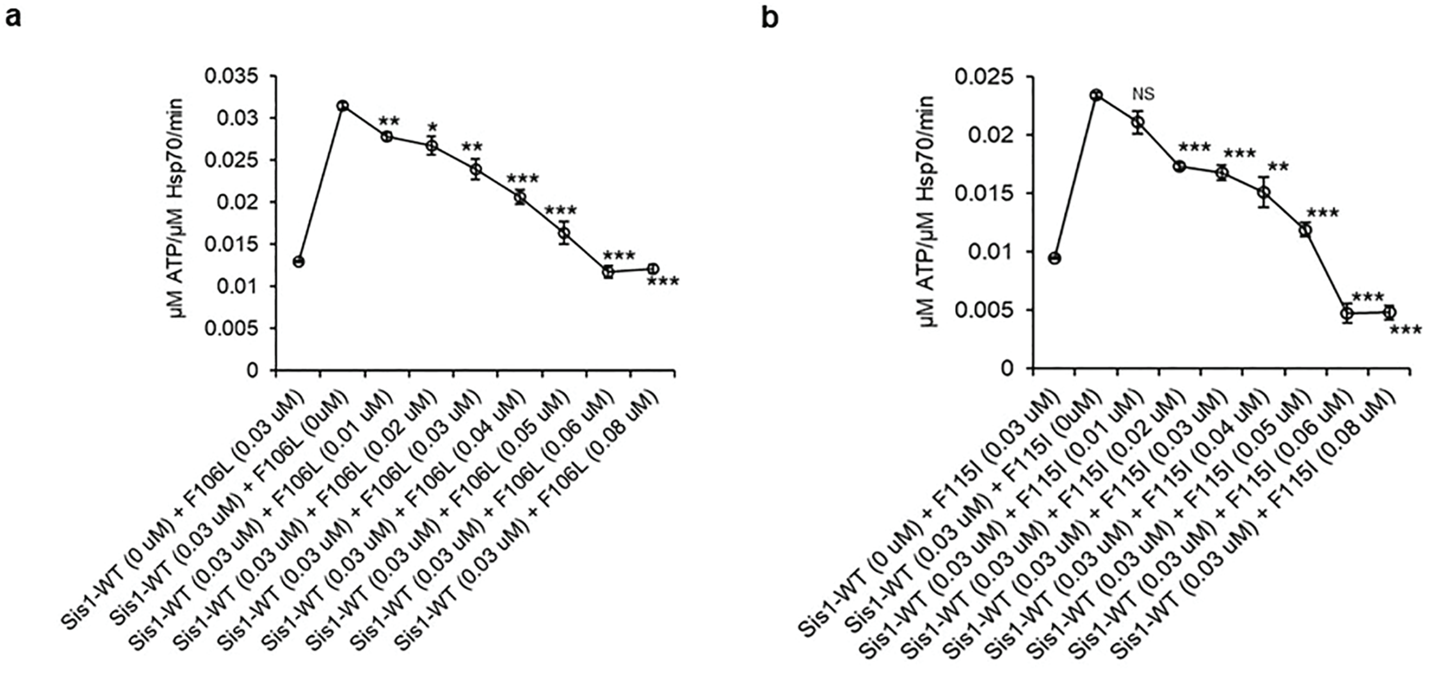

Supplementary Fig. 7. **LGMDD1 G/F domain mutants inhibit Sis1-WT induced ATPase activity of Ssa1**. Stimulation of Ssa1 ATPase activity in the presence of Rnq1 seeds formed at 25°C. Sis1-mutants (Sis1-F106L (a), or Sis1-F115I (b)) (0-0.08 µM) were titrated with Sis1-WT (0.03 µM) in the presence of Ssa1 (1 µM) and ATP (1 mM). The fraction of ATP converted to ADP was determined at 30 minutes. Data represented as mean ± SEM, n=3 biologically independent samples. For (a) and (b); values of the increasing concentration of LGMDD1 mutants (0.01-0.08 µM) were compared with Sis1-WT (0.03 µM) alone. For (a) (top to bottom); ***p*<0.006, **p*<0.015, ***p*<0.004, ****p*<0.0003, ****p*<0.0004, ****p*<2.12e-05, ****p*<9.6e-06. For (b) (top to bottom); NS=0.08 (non-significant), ****p*<7.52e-05, ****p*<0.0007, ***p*<0.003, ****p*<5.99e-05, ****p*<2.99e-05, ****p*<1.05e-05. *p*-values are reported for unpaired, two-sided *t*-test. Source data are provided as a Source data file.

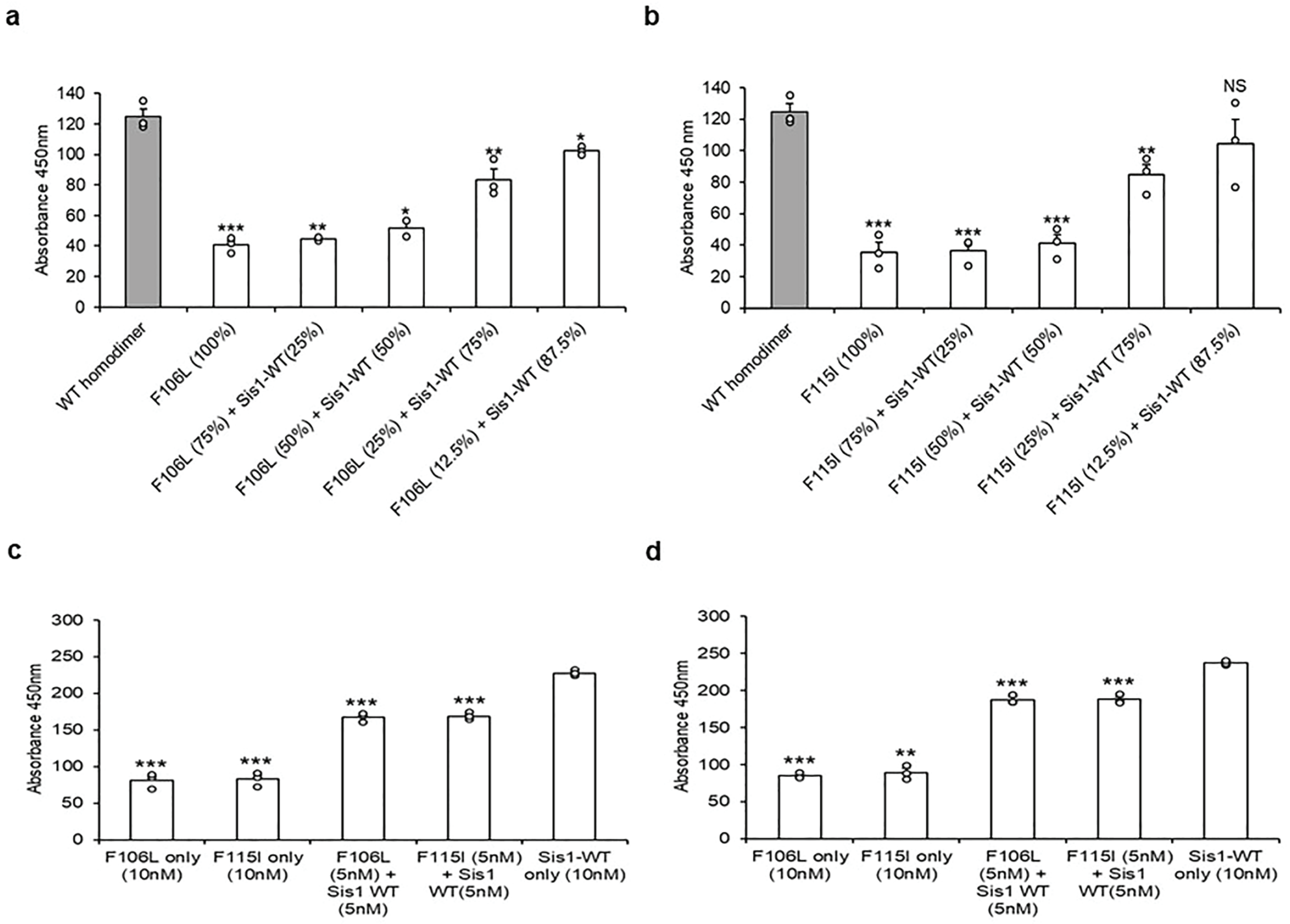

Supplementary Fig. 8. **LGMDD1 G/F domain mutants inhibit the ability of wild-type Sis1 to dimerize and bind substrates**. Sis1-WT was used in one row of the microtiter plate (as homodimer control, black) and the rest rows had Sis1-WT mixed with different concentrations of mutants (a) F106L, and (b) F115I. Data represented as mean ± SEM, n=3 biologically independent samples. Mutants mixed with either mutants were compared with Sis1-WT homodimer (grey bar). For (a) (left to right); ****p*<0.0001, ***p*<0.008, **p*<0.013, ***p*<0.009, **p*<0.016. For (b) (left to right); ****p*<0.0004, ****p*<0.0002, ****p*<0.0004, ***p*<0.009, NS=0.288 (non-significant). *p*-values are reported for unpaired, two-sided *t*-test. Binding of purified Sis1-WT (10 mM), Sis1-F106L (10 mM), Sis1-F115I (10 mM), or equimolar Sis1-WT and mutants (5 mM each) to denatured Rnq1 (c) and luciferase (d). Here, values were compared with Sis1-WT only. For (c) (left to right); ****p*<2.24e-05, ****p*<1.78e-05, ****p*<0.0001, ****p*<8.55e-05. For (d) (left to right); ****p*<0.0005, ***p*<0.003, ****p*<0.0002, ****p*<0.0002. *p*-values are reported for unpaired, two-sided *t*-test. Source data are provided as a Source data file.

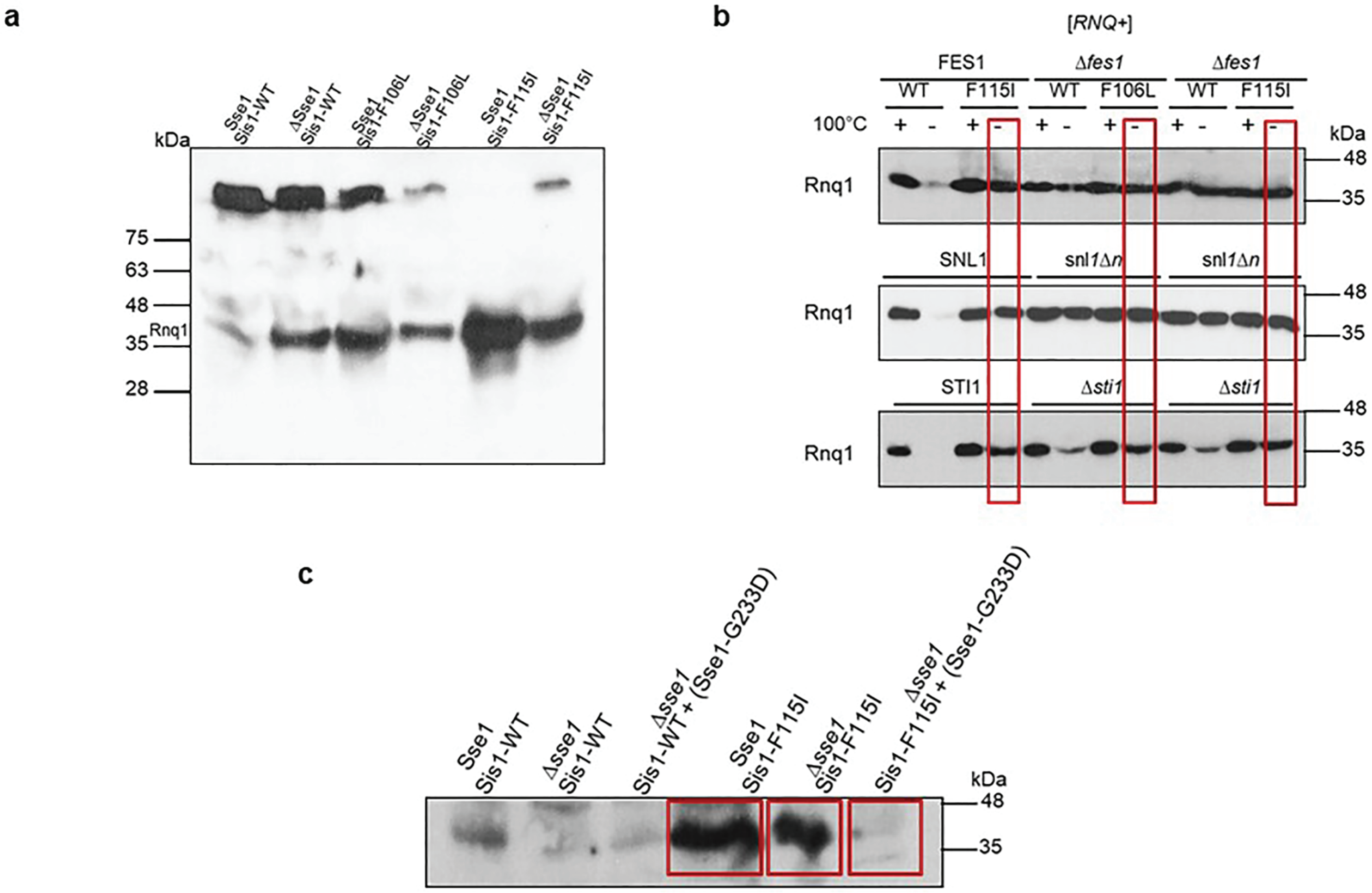

Supplementary Fig. 9. **Deletion Sse1 function partially rescues prion propagation of [*RNQ+*]**. (a) Boiled gel assays show the presence of more aggregated and less soluble Rnq1 protein in Sse1 cells expressing Sis1-WT, indicating proper [*RNQ+*] propagation. Prion propagation was reduced in Sse1 cells with the expression of LGMDD1 mutants, however, prion propagation was partially restored with the loss of Sse1 in these cells. (b) Deletion of the other NEF's did not rescue the propagation of [*RNQ+*]. (c) Sse1 mutant rescue prion propagation of [*RNQ+*]. Boiled gel assay showed less soluble Rnq1 protein (just like in Sse1 cells harboring Sis1-WT) in Δ*sse1* cells co-expressing LGMDD1 mutant and Sse1-G233D, indicating restoration of [*RNQ+*] prion propagation. For (a)-(c); experiments were done in triplicates, representative images shown.

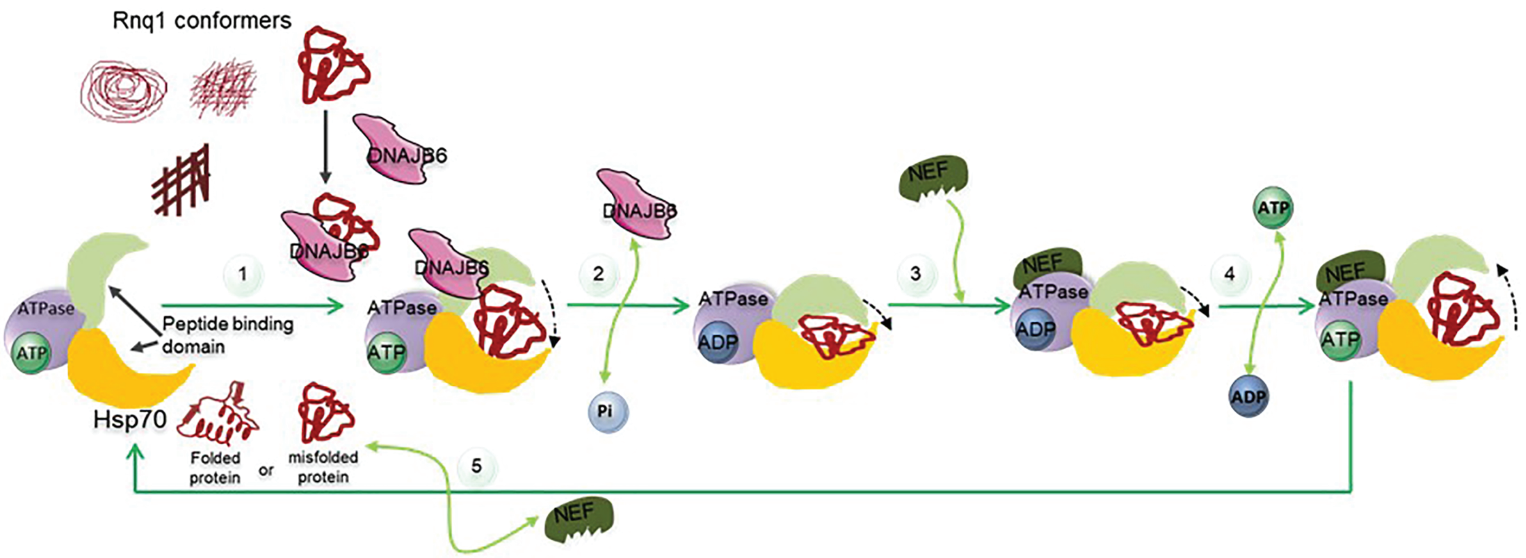

Supplementary Fig. 10. Cartoon depicting the normal processing of one of the conformers (substrate) of an aggregated protein, mediated by DNAJB6 (Hsp40) through the Hsp70 ATPase cycle. The green arrows throughout the cycle indicate normal functioning.

Primer name	Sequence
Sis1-F106L	5'-gcatcctcattacttaacgcatgtcctccggaga-3'<br>5'-tctccggaggacatgcgttaagtaatgaggatgc-3'
Sis1-N108L	5'-tgaaaaaatattgaaagcatcctctagactgaacgcatgtcctccggaga-3'<br>5'-tctccggaggacatgcgttcagtctagaggatgctttcaatattttttca-3'
Sis1-D110Δ	5'-gaggacatgcgttcagtaatgaggctttcaatattttttcacaattc-3'<br>5'-gaattgtgaaaaaatattgaaagcctcattactgaacgcatgtcctc-3'
Sis1-F115I	5'-ccgccaaagaattgtgaaataatattgaaagcatcctcattactg-3'<br>5'-cagtaatgaggatgctttcaatattatttcacaattctttggcgg-3'
Sis1-ΔDD	5'-ttgtaaaataaatggtcaaggagacaaaact-3'<br>5'-ccatttattttacaatcaaattacctctctga-3'
5 Sis1 BamHI	5'-atGGATCCatggtcaaggagacaaaact-3'
3 Sis1 SalI	5'-tgGTCGACttaaaaattttcatctatag-3'
5 Ssa1 BamHI	5'-atGGATCCatgtcaaaagctgtcggtat-3'
3 Ssa1 NotI	5'-tgGCGGCCGCttaatcaacttcttcaac-3'
snl1Δ	5'-tcaatcctgtttgttggtgaaaaaatagcaccagaagggcaattgtacgtttccgtaatg cgtacgctgcaggtcgac-3'<br>5'-atgagtgcagcctatgaattcggcaagagccgttatctataaactaaaaatacaaacttaatcgatgaattcgagctcg-3'
fes1Δ	5'-gaaagccattacctttcaacgaaagagtaaaatagaaaaaaaaacacatacataactatgcgtacgctgcaggtcgac-3'<br>5'-cggttaaagataatatgaaatggtgaatgtaatatcattttatttctacggacgtaatcaatcgatgaattcgagctcg-3'
sti1Δ	5'-aaagtctgctcccaaattcctcactgtagctactaaaacaacctatacgcaagaaagatgcgtacgctgcaggtcgac-3'<br>5'-tatgaaaaagcagtaaaaaaagaattcaagataataaagttatatttcgtattatttttaatcgatgaattcgagctcg-3'

Supplementary Table 1: **Primers used in this study**.

Supplementary Figure References

1. Bardill, J. P. & True, H. L. Heterologous prion interactions are altered by mutations in the prion protein Rnq1p. *J Mol Biol* **388**, 583–596 (2009).

2. Vitrenko, Y. A., Pavon, M. E., Stone, S. I. & Liebman, S. W. Propagation of the [PIN+] prion by fragments of Rnq1 fused to GFP. *Curr Genet* **51**, 309–319 (2007).
