# Supplementary material for: Disease-associated mutations within the yeast DNAJB6 homolog Sis1 slow conformer-specific substrate processing and can be corrected by the modulation of nucleotide exchange factors
Source: Nat Commun. 2022 Aug 5;13:4570. doi: 10.1038/s41467-022-32318-9 (PMC9355953; doi:10.1038/s41467-022-32318-9)
Supplement: Supplementary file 3 — Reporting Summary [file 41467_2022_32318_MOESM3_ESM.pdf]

nature portfolio

Corresponding author(s): Heather L. True

Last updated by author(s): Jul 1, 2022

Reporting Summary

Nature Portfolio wishes to improve the reproducibility of the work that we publish. This form provides structure for consistency and transparency in reporting. For further information on Nature Portfolio policies, see our Editorial Policies and the Editorial Policy Checklist.

Statistics

For all statistical analyses, confirm that the following items are present in the figure legend, table legend, main text, or Methods section.

n/a	Confirmed	
☐	☒	The exact sample size (*n*) for each experimental group/condition, given as a discrete number and unit of measurement
☐	☒	A statement on whether measurements were taken from distinct samples or whether the same sample was measured repeatedly
☐	☒	The statistical test(s) used AND whether they are one- or two-sided<br>*Only common tests should be described solely by name; describe more complex techniques in the Methods section.*
☒	☐	A description of all covariates tested
☒	☐	A description of any assumptions or corrections, such as tests of normality and adjustment for multiple comparisons
☐	☒	A full description of the statistical parameters including central tendency (e.g. means) or other basic estimates (e.g. regression coefficient) AND variation (e.g. standard deviation) or associated estimates of uncertainty (e.g. confidence intervals)
☐	☒	For null hypothesis testing, the test statistic (e.g. *F*, *t*, *r*) with confidence intervals, effect sizes, degrees of freedom and *P* value noted<br>*Give P values as exact values whenever suitable.*
☒	☐	For Bayesian analysis, information on the choice of priors and Markov chain Monte Carlo settings
☒	☐	For hierarchical and complex designs, identification of the appropriate level for tests and full reporting of outcomes
☒	☐	Estimates of effect sizes (e.g. Cohen's *d*, Pearson's *r*), indicating how they were calculated

Our web collection on statistics for biologists contains articles on many of the points above.

Software and code

Policy information about availability of computer code

Data collection	No novel software was used for data collection.
Data analysis	GraphPad Prism (v 8, https://www.graphpad.com/scientific-software/prism/), Microsoft Excel 2016 (https://www.https://www.microsoft.com/en-us/microsoft-365/microsoft-office), AMT ImageCapture Engine acquisition software (version V701).

For manuscripts utilizing custom algorithms or software that are central to the research but not yet described in published literature, software must be made available to editors and reviewers. We strongly encourage code deposition in a community repository (e.g. GitHub). See the Nature Portfolio guidelines for submitting code & software for further information.

Data

Policy information about availability of data

All manuscripts must include a data availability statement. This statement should provide the following information, where applicable:

- Accession codes, unique identifiers, or web links for publicly available datasets
- A description of any restrictions on data availability
- For clinical datasets or third party data, please ensure that the statement adheres to our policy

Source data for figures (including supplementary figures) are included in source data file.

Field-specific reporting

Please select the one below that is the best fit for your research. If you are not sure, read the appropriate sections before making your selection.

☒ Life sciences ☐ Behavioural & social sciences ☐ Ecological, evolutionary & environmental sciences

For a reference copy of the document with all sections, see nature.com/documents/nr-reporting-summary-flat.pdf

Life sciences study design

All studies must disclose on these points even when the disclosure is negative.

Sample size	No statistical methods were used to predetermine sample size. Biochemical experiments were performed in triplicate on biologically independent samples as per commonly accepted field standards (Methods Enzymol 2010;470:709-34, Methods Enzymol 1999;309:274-84, Methods Enzymol. 2002;351:442-53, Methods Enzymol 2010;470:681-93) and to enable statistical analysis (e.g. calculation of mean and SEM).
Data exclusions	No data were excluded.
Replication	All experiments were replicated at least three times. Attempts at replication were successful.
Randomization	Not applicable. Experiments were performed comparing strains/proteins that were identical except for the specific experimental treatment/mutants tested.
Blinding	Not applicable. Experiments were performed comparing various treatments on otherwise comparable samples and as such it was necessary for the researchers to be aware of the treatment applied (e.g. transformation/site-directed mutagenesis/heat exposure/etc.). Appropriate cellular and biochemical controls were included in each experimental replication and results reported are predominantly quantitative in nature.

Reporting for specific materials, systems and methods

We require information from authors about some types of materials, experimental systems and methods used in many studies. Here, indicate whether each material, system or method listed is relevant to your study. If you are not sure if a list item applies to your research, read the appropriate section before selecting a response.

Materials & experimental systems

n/a	Involved in the study	
☐	☒	Antibodies
☐	☒	Eukaryotic cell lines
☒	☐	Palaeontology and archaeology
☒	☐	Animals and other organisms
☒	☐	Human research participants
☒	☐	Clinical data
☒	☐	Dual use research of concern

Methods

n/a	Involved in the study	
☒	☐	ChIP-seq
☒	☐	Flow cytometry
☒	☐	MRI-based neuroimaging

Antibodies

Antibodies used	Sis1 antibody (CosmoBio, Cat#COP-080051), Hsp70 (Ssa1) antibody (Abcam, Cat#ab-5439), 6X-Histidine Tag antibody (clone-4A12E4) (Invitrogen, Cat#37-2900), Rnq1 antibody (Cocalico Biologicals; p35367 (CTD 148-405). Donkey anti-rabbit (cat#AP182P), Mouse anti-rabbit (cat#MAB201P), Rabbit anti-mouse (Cat#AP160P) HRP-conjugated secondary antibodies were purchased from Millipore-Sigma. Goat anti-mouse IgG [H+L] (cat#62-6520) HRP-conjugated secondary antibody was purchased from Thermo-Fisher Scientific.
Validation	All antibodies have been previously used for the application and species described here. Sis1 antibody has been validated for use with recombinant protein (https://www.cosmobiousa.com/content/document/cosmo-bio-ltd/cop-cop-080051_anti-sis1dnaj-pab_datasheet.pdf), Hsp70 (Ssa1) antibody has been validated (https://www.abcam.com/hsp70-antibody-3a3-ab5439.html?productWallTab=ShowAll), 6X-Histidine Tag antibody has been validated (https://www.thermofisher.com/antibody/product/6x-His-Tag-Antibody-clone-4A12E4-Monoclonal/37-2900), Rnq1 antibody is custom made polyclonal antibody from Cocalico Biologicals against CTD (148-405 aa) of Rnq1 protein and the same has been validated previously.

Eukaryotic cell lines

Policy information about cell lines

Cell line source(s)	Yeast parent cells from Susan Leibman lab.

Authentication	Phenotypic testing for relevant markers.
Mycoplasma contamination	Not applicable.
Commonly misidentified lines (See ICLAC register)	Not applicable.
